# Effect of Antibiotic, Multivitamin and Iron Preparations on Color Stability of Pediatric Restorative Materials: An In Vitro Study

**DOI:** 10.3390/jfb17100521

**Published:** 2026-10-16

**Authors:** Mesut Kayğusuz, Güldeste Aydın, Mehmet Sinan Doğan, Mehmet Emin Doğan

**Affiliations:** 1 Department of Pediatric Dentistry, Faculty of Dentistry, Harran University, Sanliurfa 63290, Türkiye; mesutkaygusuz@harran.edu.tr (M.K.); drmsdogan@harran.edu.tr (M.S.D.); 2 Department of Dentomaxillofacial Radiology, Faculty of Dentistry, Harran University, Sanliurfa 63290, Türkiye; medogan@harran.edu.tr

**Keywords:** pediatric dentistry, color stability, pediatric liquid medications, dental restorative materials

## Abstract

**Background/Objectives:** This in vitro study compared the color stability of four pediatric restorative materials after exposure to three pediatric liquid medications in a 4 × 3 factorial design. **Methods:** Sixty disc specimens (10.0 × 2.0 mm) were prepared from a composite resin (Charisma Smart), a compomer (Nova Compomer), a giomer (Beautifil Kids SA), and a high-viscosity glass ionomer cement (HVGIC; Equia Forte HT with Equia Forte Coat) (*n* = 15 per material) and allocated to antibiotic, multivitamin syrup, and iron preparation subgroups (*n* = 5); the specimens were immersed for 2 min twice daily for 7 days. ΔE_00_ values were calculated from VITA Easyshade V measurements at baseline and after exposure and were analyzed using two-way ANOVA with Bonferroni-adjusted comparisons. **Results:** Restorative material (F(3,48) = 11.221, *p* < 0.001) and medication type (F(2,48) = 6.937, *p* = 0.002) had significant main effects, whereas their interaction was not significant (F(6,48) = 1.676, *p* = 0.147). HVGIC showed significantly greater color change than the other materials (*p* < 0.05). Exposure to the antibiotic suspension was associated with greater discoloration than exposure to the iron preparation (*p* = 0.002). The giomer had the lowest mean ΔE_00_, but it did not differ significantly from the compomer and composite resin. **Conclusions:** ΔE_00_ differed significantly among the restorative products and among the three medication exposure conditions, with no significant interaction. Because no unexposed control group was included, these differences reflect comparisons between exposure conditions rather than the effect of the medications alone. These in vitro findings are product-specific and require clinical confirmation.

## 1. Introduction

Children frequently receive liquid medications, often repeatedly or over prolonged periods, and these preparations contain acidic vehicles, sugars, thickeners, and colorants that remain in contact with teeth and restorations. Tooth-colored restorations placed in primary and young permanent teeth are therefore repeatedly exposed to chemically diverse liquids, and their color stability influences how long a restoration remains acceptable and when it must be replaced. Consequently, increasing attention has been directed toward restorative materials designed to reproduce the structural and aesthetic characteristics of natural teeth [1,2]. Materials such as glass ionomer cements, composite resins, compomers, and giomers, which can mimic the natural color of teeth, allow both anterior and posterior teeth to be restored, enabling the rehabilitation of defective teeth [3,4]. Composite resins emerged following the development of Bis-GMA and the subsequent addition of an inorganic filler [5,6]. Conventional glass ionomer cements (GICs) play an important role in pediatric restorative dentistry because of their ability to chemically bond to dental hard tissues and release fluoride. However, their clinical performance may be limited by relatively low mechanical durability, suboptimal aesthetic properties, and susceptibility to moisture. To address these limitations, modifications in glass ionomer technology have led to the development of high-viscosity GICs with increased powder-to-liquid ratios and altered particle characteristics. Conversely, restorative materials with superior physical properties may release less fluoride, potentially limiting their caries-preventive benefit. Compomers, on the other hand, were developed by mixing a fluoride-containing glass filler with a polyacid-modified resin. However, compomers release less fluoride, and for a shorter duration, than glass ionomers [7], and they have weaker physical properties than resin composites. Giomers are resin-based restorative materials incorporating pre-reacted glass (PRG) fillers, which enable fluoride release from the material. Their composition was designed to integrate the fluoride-releasing potential associated with glass ionomer systems with the favorable mechanical characteristics of resin-based restoratives. Furthermore, owing to their ion-releasing surface pre-reacted glass (S-PRG) filler, giomers may neutralize acidic challenges and thus help to prevent secondary caries [8,9,10].

Maintaining color over time is an important consideration when evaluating the clinical performance of restorative materials [11]. Discoloration of restorative materials can result from material-related characteristics as well as exposure to the oral environment. Factors associated with the material itself and its clinical handling, including optical behavior, resin and matrix composition, degree of polymerization, and surface characteristics, may influence its susceptibility to color alteration. In addition, exposure to biofilm, dietary chromogens, acidic conditions, and medications may further modify the color of a restoration over time [12,13,14].

Syrups and suspensions, which are used to facilitate medication administration in pediatric patients, play a critical role, particularly in the treatment of children who have difficulty swallowing. However, the buffering agents, acidic ingredients with low pH values, and coloring pigments present in the formulations of these liquid medications, although they form part of the treatment, also carry the risk of causing undesirable effects on the surfaces of teeth and dental restorations [15].

Iron-containing preparations are among the most commonly used supplements during childhood. Iron supplements are used in children to prevent hematological disorders, support brain development, and increase attention span [16]. Although iron supplements support systemic health, they can cause aesthetic problems in oral tissues and dental restorations [11]. The black precipitates formed when iron ions interact with sulfur compounds in the mouth are not merely superficial stains; they reflect a complex process that adversely affects the internal structure and aesthetic appearance of restorative materials through oxidation and ionic changes [13].

Multivitamins, which are used as dietary supplements combining various vitamins to provide the body with additional nutrients and to complement and fortify the diet, are administered to children in syrup form. The physical and chemical characteristics of liquid medications may contribute to their interaction with dental tissues and restorative materials. In particular, a viscous formulation may prolong contact with these surfaces and thereby increase the potential for discoloration. Moreover, many liquid medications have an acidic pH, which is related to formulation requirements such as product stability and drug performance. The acidic nature of multivitamin preparations may adversely affect the surface integrity of restorative materials, leading to increased surface roughness and, consequently, to more severe staining due to pigment accumulation on the surface. The final factor in multivitamin-related discoloration is the colorants used [2,11,16].

Antibiotic suspensions, widely prescribed in pediatric practice for common bacterial infections, have also been shown to compromise the color stability of restorative materials because of their acidic vehicles, high sugar content, thickeners, and synthetic colorants [17,18,19]. Previous studies have demonstrated that amoxicillin suspensions induce noticeable discoloration in resin composites and glass ionomer cements [20], whereas amoxicillin and azithromycin formulations produced color shifts exceeding clinically perceptible thresholds in simulated oral environments [21]. Similarly, exposure to amoxicillin/clavulanate, clarithromycin, and cephalexin suspensions resulted in perceptible chromatic shifts across various restorative substrates [11]. Consequently, evaluating the staining potential of frequently administered antibiotic suspensions alongside nutritional supplements is essential for predicting the clinical aesthetic longevity of pediatric restorations.

Color assessment of dental structures and restorative materials can be performed using both visual approaches and various instrument-based techniques. These include shade guides, colorimeters, spectroradiometers, digital imaging systems, and spectrophotometric methods. Spectrophotometric evaluation enables quantitative characterization of the light reflected from or transmitted through a material across different wavelengths [2].

Because color perception varies between observers and is open to personal interpretation, the need for a more standardized and reproducible evaluation system has increased. In this context, the CIE L∗a∗b∗ (International Commission on Illumination) system, which expresses color numerically using three fundamental coordinates, was developed. In this system, the L∗ parameter represents the level of lightness or brightness, a∗ represents the position on the red–green axis, and b∗ represents the position on the yellow–blue axis. These data, obtained using a spectrophotometer, are plotted on a three-dimensional graph to determine the material’s color coordinates. Today, the CIE L∗a∗b∗ system is one of the most reliable and standardized methods used in dental research to quantitatively express color changes [22,23].

Although the effects of various medications on restorative materials have been extensively investigated in the literature, data on the color stability of giomers exposed to specific medications frequently used in the pediatric population for chronic or supportive purposes remain limited [3,8,11,20,23,24,25,26]. Moreover, despite previous studies investigating drug-induced color changes, evidence from simultaneous comparisons of specific restorative products exposed to antibiotics, multivitamins, and iron preparations under the same standardized protocol—and, in particular, the separate and interactive effects of the restorative product and the type of medication on color change—remains insufficient. This in vitro study aimed to compare the color changes observed in four restorative products after repeated exposure to pediatric iron, antibiotic, and multivitamin medications. The null hypotheses were that restorative material type and pediatric medication type would have no significant main effects on ΔE_00_ and that there would be no significant interaction between these two factors.

## 2. Materials and Methods

In this study, three liquid medications frequently prescribed to children and four restorative materials commonly used in pediatric dentistry were evaluated (Table 1 and Table 2). For standardization, shade A2 was selected for the restorative materials.

### 2.1. Preparation of Specimens

**Figure 1:**
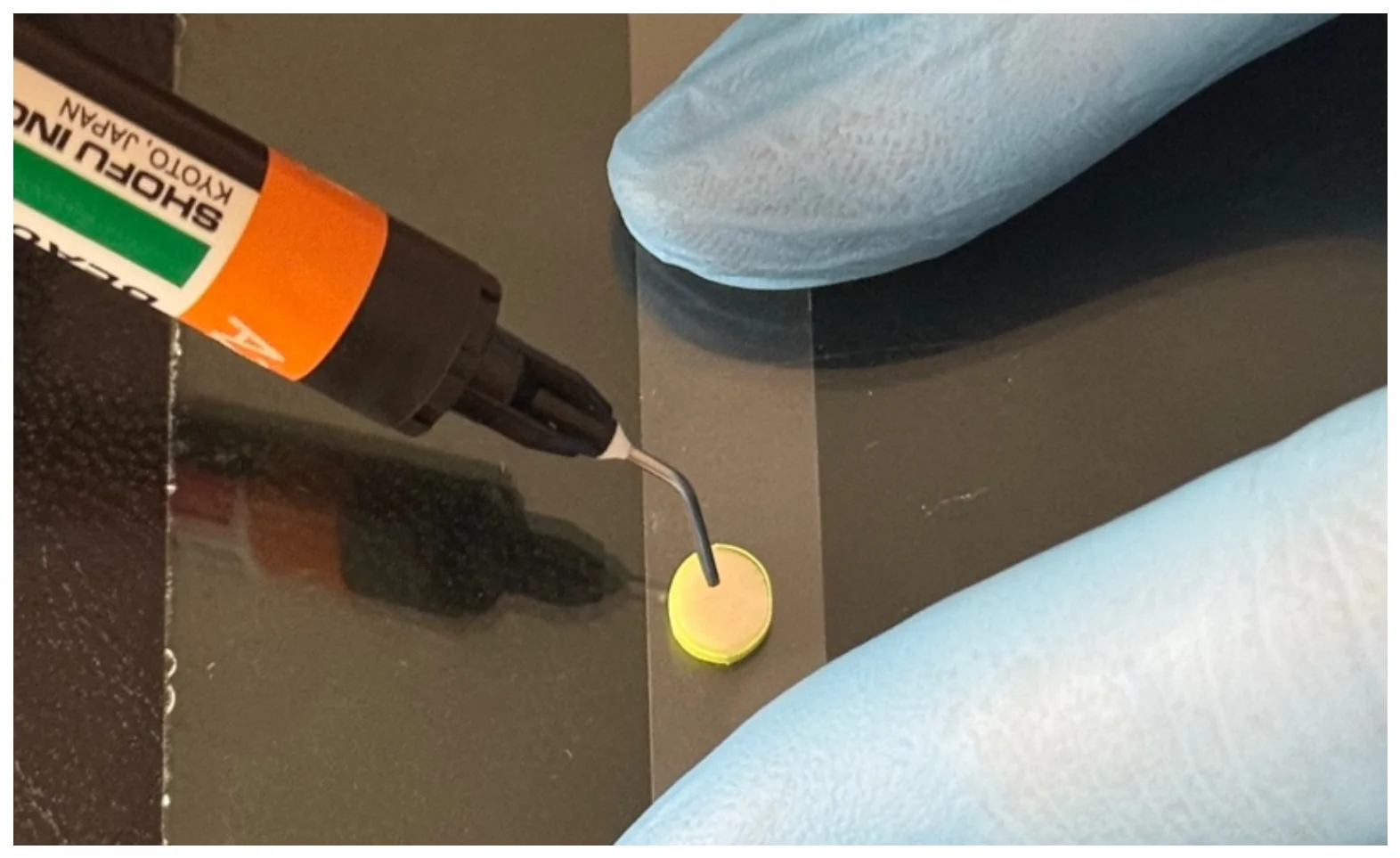
Preparation of the material discs.

All specimens were prepared using standardized, custom-made circular polytetrafluoroethylene (PTFE, Teflon) split molds (10.0 mm inner diameter and 2.0 mm depth) to ensure uniform dimensions and permit specimen retrieval without introducing microcracks or surface defects. A total of 60 specimens were prepared, comprising 15 specimens for each of the four restorative material groups, in strict accordance with the manufacturers’ instructions. Photopolymerization of the composite resin, giomer, and compomer specimens was carried out for 20 s on both the top and bottom surfaces using a calibrated light-emitting diode (LED) device (Woodpecker LED.D, Guilin Woodpecker Medical Instrument Co., Ltd., Guilin, China; wavelength range: 420–480 nm; optical tip diameter: 8.0 mm; irradiance verified with a digital radiometer at 1000–1200 mW/cm^2^). Each uncured specimen was covered with a transparent polyester matrix strip (Mylar strip) and compressed with a microscope glass slide under standardized manual pressure to achieve uniform thickness, eliminate flash, and prevent oxygen-inhibited layer formation. The light-curing tip was positioned perpendicular to, and in direct contact with, the surface of the Mylar strip (representing a standardized distance of approximately 0.05 mm, defined by the matrix strip thickness). The high-viscosity glass ionomer cement (Equia Forte HT) specimens were prepared by mixing the capsules in an amalgamator (TPC D-650 Digital Amalgamator, TPC Advanced Technology, Inc., City of Industry, CA, USA) for 10 s, placing them into the molds using an applier, and allowing them to set for 2 min and 30 s from the start of mixing (initial setting time according to the manufacturer). Following setting, the specimens were carefully retrieved from the split molds (Figure 1).

Following demolding, all specimens were systematically inspected under dental loupes at 3.5× magnification with continuous coaxial LED illumination to screen for internal voids, structural defects, margin chipping, or surface irregularities; specimen dimensions were also confirmed using a digital caliper, and any specimen that was not structurally uniform was immediately excluded and replaced. Aluminum oxide polishing discs (coarse, medium, fine, and super-fine Sof-Lex Polishing Discs, 3M ESPE, St. Paul, MN, USA) were used for the finishing and polishing of all specimens. Polishing was performed sequentially with Sof-Lex discs attached to a low-speed micromotor handpiece operating at 10,000 rpm (coarse and medium grits) and 15,000 rpm (fine and super-fine grits) using light, intermittent pressure for 15 s per step. After completion of the polishing protocol, the dimensions (diameter and thickness) of all specimens—including the high-viscosity glass ionomer specimens—were re-measured with a digital caliper (0.01 mm precision) to confirm that standard dimensions were maintained (10.0 ± 0.05 mm diameter and 2.0 ± 0.05 mm thickness) and to discard any specimen exhibiting measurable abrasion or uneven reduction. Each specimen was then thoroughly rinsed under running distilled water for 10 s to remove surface residues. After drying, and in accordance with the manufacturer’s protocol, a protective resin coating (Equia Forte Coat, GC Corporation, Tokyo, Japan) was applied to the surfaces of the HVGIC specimens only, using a single-use microbrush, and was polymerized for 20 s using an LED device. To eliminate inter-operator variability and ensure strict standardization throughout the study, all specimen preparation, curing, and finishing/polishing procedures were performed by a single calibrated operator under identical laboratory conditions. All specimens were stored in distilled water at 37 °C for 24 h to allow post-curing of the resin-based materials and continued maturation of the glass ionomer.

### 2.2. Allocation of Specimens to Subgroups

A total of 60 disc-shaped specimens were prepared, comprising 15 specimens for each of the four restorative material groups (composite resin, compomer, giomer, and high-viscosity glass ionomer cement). The specimens within each material group were then randomly and equally divided into three subgroups (*n* = 5): Augmentin^®^, Polivit^®^, and Ferro Sanol B^®^. The sample size was evaluated using G*Power software (Version 3.1.9.7, Heinrich Heine University, Düsseldorf, Germany) for a 4×3 factorial two-way ANOVA design. Based on α=0.05, a target statistical power $\left(1-\beta \right)=0.80$, a large effect size $\left(f=0.45\right)$, and k=12 groups, the minimum total sample size required to detect the main effect was calculated as N=59 for the restorative material (numerator df=3) and N=52 for the medication type (numerator df=2). Accordingly, the total sample of 60 specimens (n=5 per subgroup) provided adequate statistical power (>80%) to test both primary main effects. However, detecting the material × medication interaction effect (df=6) under identical parameters required a larger sample (N≥75), yielding a statistical power of approximately 68% for the interaction term at the present sample size.

### 2.3. Color Change Measurements

The first color measurement of the specimens after polymerization was performed using a contact spectrophotometer (VITA Easyshade^®^ V, VITA Zahnfabrik GmbH & Co. KG, Bad Säckingen, Germany), ensuring that each group was tested under identical conditions. The spectrophotometer was calibrated prior to the measurements, and each specimen was positioned centrally. The measurements were performed against a gray background with standardized position, angle, and ambient lighting. The color coordinates of the specimens were determined spectrophotometrically using the CIELAB color system.

Following baseline color measurements, the specimens—previously stored in distilled water—were subjected to an immersion protocol simulating a clinical therapeutic course. The pediatric medications evaluated comprised an antibiotic (Augmentin^®^ oral suspension), a multivitamin syrup (Polivit^®^), and an iron preparation (Ferro Sanol B^®^ drops). All medications were prepared and handled in strict accordance with the manufacturers’ instructions. Augmentin^®^ dry powder was reconstituted with distilled water to the indicated volume, shaken thoroughly to obtain a homogeneous suspension, and stored at 4 °C between cycles. Ready-to-use liquid formulations (Polivit^®^ and Ferro Sanol B^®^) were stored at a measured room temperature (23 ± 1 °C), protected from light, and shaken vigorously before each immersion. To closely replicate the standard clinical single-dose administration in pediatric patients, each specimen was individually immersed in a sterile, airtight vial containing 5 mL of the respective undiluted medication, selected to reflect the direct, transient contact of pediatric syrups with restorative surfaces during oral ingestion. The specimens were immersed for 2 min twice daily (at 12-h intervals) over a 7-day period. Fresh medication was dispensed for every immersion cycle to simulate newly administered doses. Following each immersion, specimens were gently rinsed with distilled water for 10 s to eliminate residual medication. To standardize the drying procedure without introducing mechanical surface artifacts, excess surface moisture was blotted with absorbent paper without rubbing or friction, and specimens were returned to distilled water at 37 °C until the subsequent cycle. The temperatures of all immersion media were verified using a calibrated digital thermometer (Versatile, VCD-800, Beijing, China). The pH values given in Table 1 were not measured in this study; they were taken from the literature [27,28].

Color was assessed using a VITA Easyshade V spectrophotometer (VITA Zahnfabrik GmbH & Co. KG, Bad Säckingen, Germany), which was calibrated according to the manufacturer’s instructions before measurement. All baseline and post-exposure measurements were performed with the same VITA Easyshade V device against the same gray background. Immediately before each measurement session, specimens were removed from distilled water and their surfaces were blotted with absorbent paper in a standardized manner. For each specimen, three consecutive readings were obtained with the probe tip held in the same position on the specimen surface without being lifted between readings, and the arithmetic mean of the three readings was used for statistical evaluation. The overall difference between baseline and post-exposure color coordinates was then calculated as ΔE_00_ using the CIEDE2000 formula:

$$\Delta E_{00}=\sqrt{{\left(\frac{\Delta L^{\prime }}{k_{L}S_{L}}\right)}^{2}+{\left(\frac{\Delta C^{\prime }}{k_{C}S_{C}}\right)}^{2}+{\left(\frac{\Delta H^{\prime }}{k_{H}S_{H}}\right)}^{2}+R_{T}\left(\frac{\Delta C^{\prime }}{k_{C}S_{C}}\right)\left(\frac{\Delta H^{\prime }}{k_{H}S_{H}}\right)}$$

Δ*L*′ denotes the lightness difference, Δ*C*′ the chroma difference, and Δ*H*′ the hue difference. *S*_L_, *S*_C_, and *S*_H_ represent the weighting functions for lightness, chroma, and hue, respectively; the parameters *k*_L_, *k*_C,_ and *k*_H_ are generally taken to be 1 under experimental conditions. *R_T_* is the rotation term indicating the interaction between chroma and hue. In this study, *k*_L_ = *k_C_* = k_H_ = 1 was assumed. In the CIE L*a*b* coordinate system, directional shifts are defined such that a negative ΔL* indicates darkening, while negative Δa* and Δb* coordinates represent chromatic shifts toward green and blue, respectively.

### 2.4. Statistical Analysis

Statistical analyses were performed using SPSS software, version 26.0 (IBM Corp., Armonk, NY, USA). The normality of the data distribution was assessed using the Shapiro–Wilk test. Homogeneity of variances was evaluated using Levene’s test (α = 0.05). Descriptive statistics are expressed as mean ± standard deviation. The experimental unit was the individual specimen (*n* = 5 per cell); the mean of the three color readings per specimen was used in the analysis. Considering the 4 × 3 factorial experimental design involving four restorative materials and three pediatric medications, total color change ($\Delta E_{00}$) was analyzed using a two-way analysis of variance (two-way ANOVA) to assess the main effects of restorative material, pediatric medication, and their interaction term. Effect sizes were reported as partial eta squared ($\eta_{p}^{2}$). Pairwise post hoc comparisons for significant main effects were performed using the Bonferroni correction, and 95% confidence intervals (95% CIs) were calculated. Because Levene’s test indicated heterogeneity of variances, a sensitivity analysis was performed in which the main effects and the interaction term were re-tested using heteroscedasticity-consistent (HC3) robust standard errors. The level of statistical significance was set at *p* < 0.05.

## 3. Results

The Shapiro–Wilk test indicated that the data were normally distributed (*p* > 0.05), whereas Levene’s test indicated significant heterogeneity of variances [F(11,48) = 2.587, *p* < 0.05]. The two-way ANOVA revealed that the main effect of restorative material on $\Delta E_{00}$ was statistically significant [$F\left(3,48\right)=11.221,p<0.001,\eta_{p}^{2}=0.412$] (Table 3). Similarly, the main effect of medication type was statistically significant [$F\left(2,48\right)=6.937,p=0.002,\eta_{p}^{2}=0.224$]. In contrast, the material × medication interaction effect was not statistically significant [$F\left(6,48\right)=1.676,p=0.147,\eta_{p}^{2}=0.173$]. In the sensitivity analysis using HC3 robust standard errors, the main effects of restorative material [F(3,48) = 6.385, *p* < 0.001] and medication type [F(2,48) = 8.033, *p* < 0.001] remained statistically significant, whereas the material × medication interaction remained non-significant [F(6,48) = 1.708, *p* = 0.140]. Data from the initial measurements (T1) are denoted as L_1_, a_1,_ and b_1_, while post-exposure measurements (T2) are denoted as L_2_, a_2,_ and b_2_.

According to the findings, the highest ΔE_00_ values following exposure to the medications were observed in the antibiotic (8.48 ± 3.92) and multivitamin syrup (6.79 ± 2.39) subgroups of the HVGIC material.

In the composite resin group, the color change values obtained after exposure to the antibiotic (4.36 ± 2.28) and the multivitamin syrup (4.35 ± 2.36) were similar, whereas a lower ΔE_00_ value (2.07 ± 1.00) was observed after exposure to the iron preparation. In the compomer group, the highest color change was observed after exposure to the antibiotic (3.78 ± 1.95).

In the giomer group, the mean color change values were 2.51 ± 2.29, 2.76 ± 0.95, and 2.34 ± 1.00 following exposure to the antibiotic, multivitamin syrup, and iron preparation, respectively, with little variation across the three medications.

When analyzed by medication type, the antibiotic suspension resulted in the highest ΔE_00_ values in both the HVGIC (8.48 ± 3.92) and composite resin (4.36 ± 2.28) materials.

**Figure 2:**
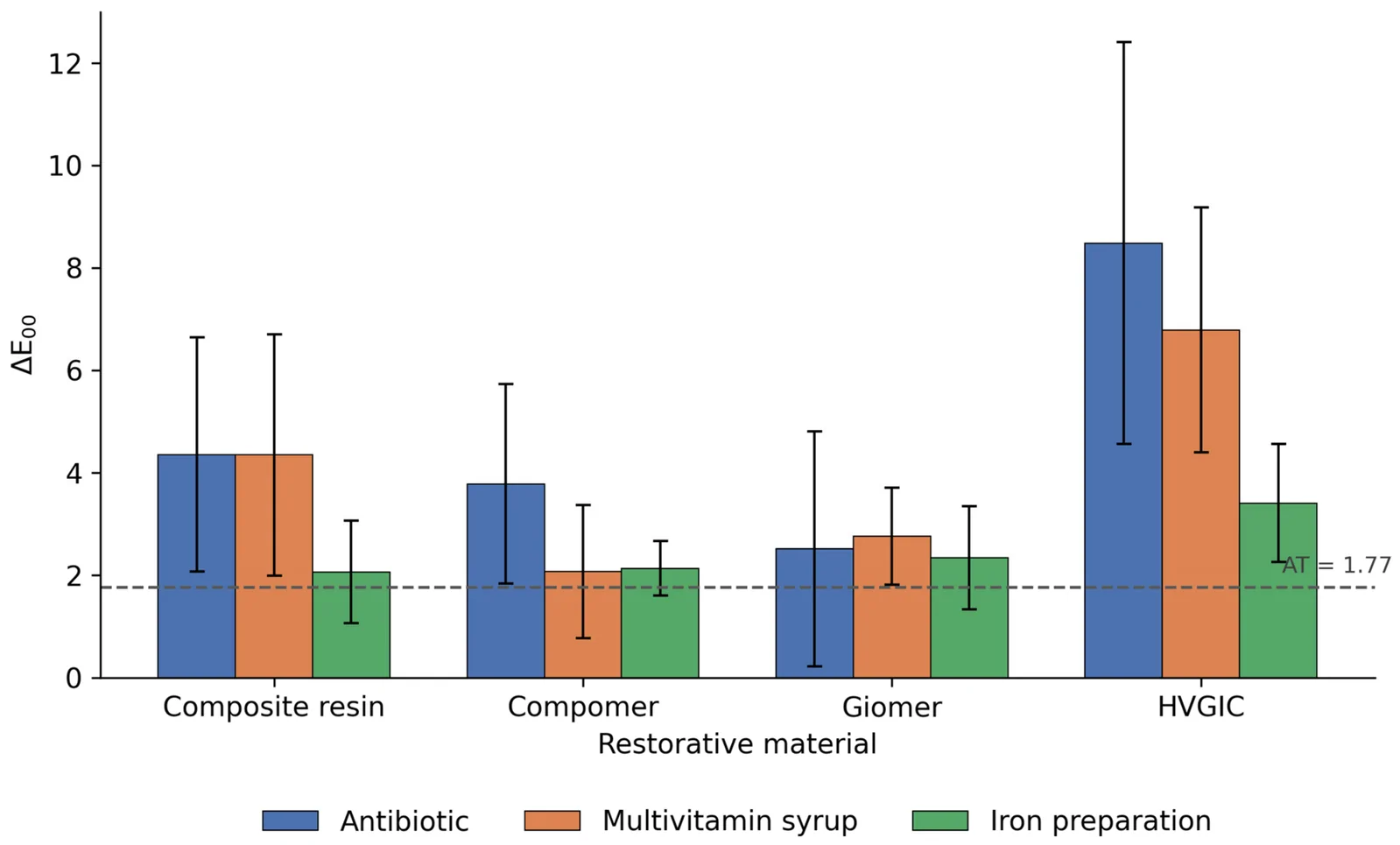
Mean $\Delta E_{00}$ color difference values with standard deviation error bars across the evaluated pediatric restorative materials after exposure to Augmentin, Polivit, and Ferro Sanol B. The horizontal dashed line denotes the acceptability threshold ($\Delta E_{00}=1.77$).

**Figure 3:**
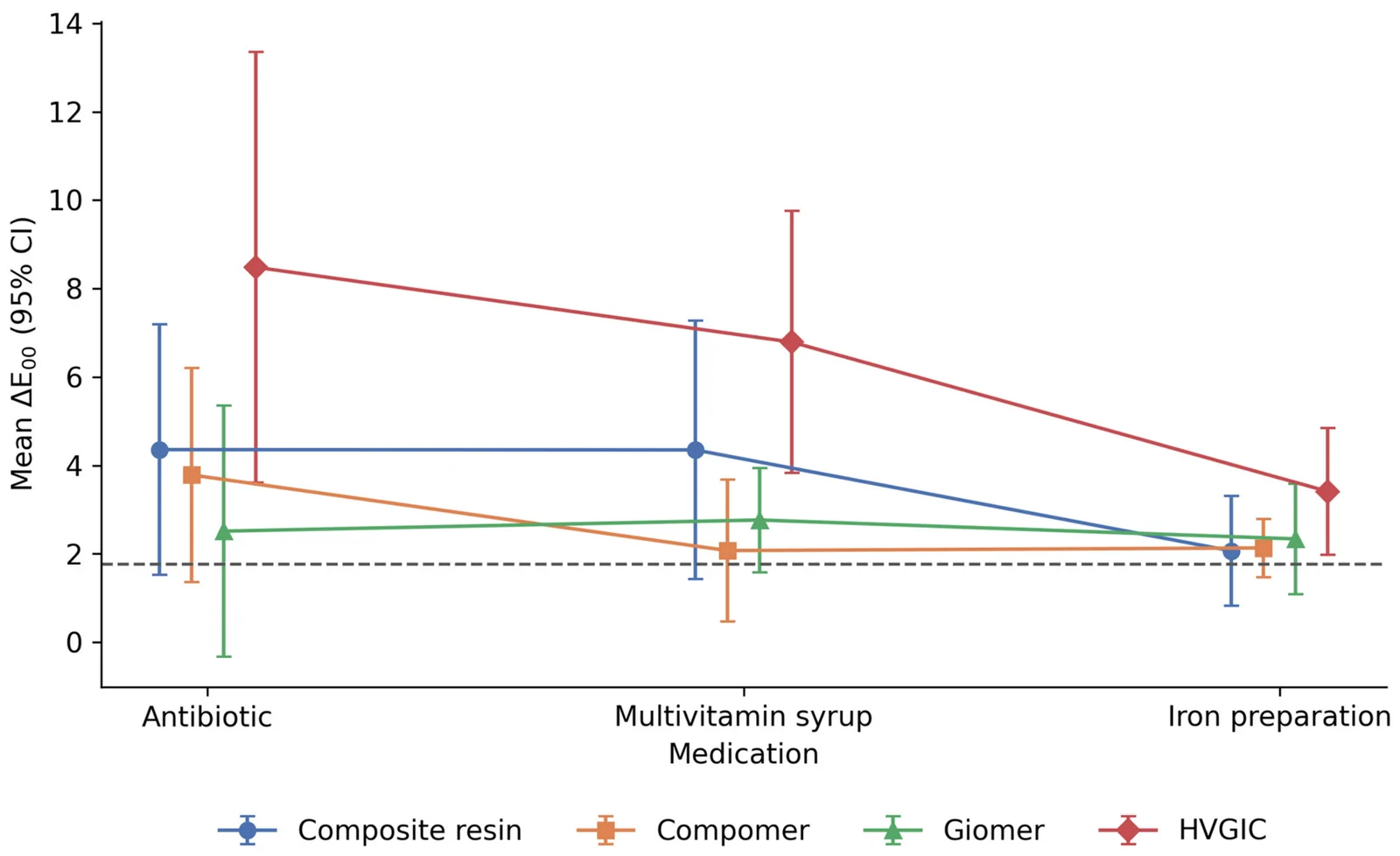
Material × medication interaction plot: mean ΔE_00_ with 95% confidence intervals (cell-specific, t-based) for each restorative material after exposure to the antibiotic, multivitamin syrup, and iron preparation. The dashed line denotes the acceptability threshold (ΔE_00_ = 1.77).

After exposure to the multivitamin syrup, the highest color change was also observed in the HVGIC group (6.79 ± 2.39); values of 4.35 ± 2.36, 2.07 ± 1.30, and 2.76 ± 0.95 were obtained in the composite resin, compomer, and giomer groups, respectively. After exposure to the iron preparation, $\Delta E_{00}$ values of 3.41 ± 1.16 were recorded in the HVGIC group, 2.34 ± 1.00 in the giomer group, 2.13 ± 0.53 in the compomer group, and 2.07 ± 1.00 in the composite resin group (Table 4, Figure 2). The material × medication interaction is illustrated in Figure 3 (cell means with 95% confidence intervals).

Pairwise comparisons with Bonferroni correction indicated that HVGIC exhibited significantly greater overall color change than composite resin (mean difference = 2.637, 95% CI: 0.646 to 4.628, p=0.004), compomer (mean difference = 3.564, 95% CI: 1.573 to 5.555, p<0.001), and giomer (mean difference = 3.689, 95% CI: 1.698 to 5.681, p<0.001) (Table 5). No significant differences were detected among the composite resin, compomer, and giomer groups (p>0.05). Regarding medication types, the antibiotic suspension differed significantly from the iron preparation (mean difference = 2.297, 95% CI: 0.743 to 3.852; p=0.002), whereas the differences between the antibiotic and the multivitamin syrup (mean difference = 0.790, 95% CI: −0.765 to 2.345, *p* = 0.641) and between the multivitamin syrup and the iron preparation (mean difference = 1.507, 95% CI: −0.047 to 3.062, *p* = 0.060) were not statistically significant (p>0.05). The pairwise comparisons between medications are given in Table 6.

Directional changes in the CIE L*a*b* coordinates (T2–T1) were also evaluated. Across all specimens, the mean changes were ΔL* = −0.18, Δa* = −0.81, and Δb* = −2.82; the mean absolute changes were |ΔL*| = 2.94, |Δa*| = 1.19, and |Δb*| = 4.22, so the largest change occurred along the b* axis. In the HVGIC–antibiotic and HVGIC–multivitamin groups, which had the highest ΔE_00_ values, the mean Δb* was −11.66 and −10.50, respectively (a decrease in b*, i.e., a shift toward blue/less yellow). The initial colors of the medications were not measured; therefore, the L*, a*, and b* changes could not be related to the original colors of the medications.

## 4. Discussion

Both the restorative product and the medication had significant main effects on ΔE_00_, so the null hypotheses for these factors were rejected. The interaction was not statistically significant and its null hypothesis could not be rejected; however, the study had only about 68% power for this term and the observed interaction effect size was large (partial η^2^ = 0.173), so the absence of a significant interaction should not be interpreted as evidence that the effects are additive. The HVGIC product differed significantly from the other materials, whereas the giomer product had the numerically lowest mean ΔE_00_ but did not differ significantly from the compomer and composite resin products.

In pediatric dentistry, color stability and anticariogenic activity are among the most important characteristics of dental restorations [11]. The ability of restorative materials to maintain their color over time is influenced by both compositional and procedural factors. Resin-matrix and filler characteristics, together with the material’s physical properties, restorative procedures, and polishing and finishing techniques, may all contribute to its color behavior [29].

Discoloration can also lead to patient dissatisfaction and impose additional financial burdens due to premature restoration replacement [30]. From a functional standpoint, staining serves as an overt manifestation of underlying material degradation: water sorption has been associated not only with chromatic alterations, but also with decreased wear resistance, compromised mechanical properties, the elution of unreacted monomers, and hydrolytic bond cleavage at the resin–filler interface [31]. Furthermore, absorbed water promotes polymer matrix plasticization, filler debonding, and reduced abrasion resistance, while concurrently facilitating pigment penetration into the matrix, thereby exacerbating extrinsic discoloration [32]. Accordingly, water acts as a carrier vehicle for exogenous chromogens, with staining susceptibility paralleling the rate of water sorption [33].

In the literature, discoloration of resin-based restorations is classified into three types: extrinsic staining characterized by plaque accumulation and superficial stains; absorption-type discoloration caused by the limited penetration of colorants into the surface or subsurface layer of the material; and intrinsic staining resulting from physicochemical reactions within the bulk of the material [33,34]. Extrinsic staining occurs through the adsorption of chromogens onto the surface and becomes more pronounced as surface roughness increases [35]; such stains can be largely removed by brushing and polishing [36]. The primary agents of this mechanism in pediatric liquid medications are synthetic colorants such as titanium dioxide, erythrosine, Sunset Yellow FCF, and Allura Red AC, which are widely used in antibiotic suspensions [17]; Red dye No. 40 is also present in a significant proportion of pediatric antimicrobial preparations [18].

Water uptake by resin-based materials may facilitate the diffusion of chromogenic molecules into the material structure, thereby contributing to discoloration. Consequently, materials with greater water sorption may be more susceptible to staining from external sources [33,37,38]. Accordingly, glass ionomer-based materials, which have relatively high water sorption, have been reported to exhibit greater color change than composites upon exposure to liquid medications [20].

The third mechanism stems from chemical interactions: in iron-containing preparations, iron ions form dark-colored insoluble precipitates [13,39]; iron exposure can also increase surface roughness, thereby indirectly facilitating stain retention [40]. In addition, certain medications can induce systemic/intrinsic discoloration in dental tissues; amoxicillin-clavulanate, minocycline, and doxycycline are among these drugs, and it has been emphasized that the color, viscosity, and pH value of the medication may play a role in staining [27,41,42,43].

Regarding the medication-specific manifestations of these mechanisms in our study, the direction of the color changes was not uniform: overall, the largest mean change occurred along the b* axis (mean |Δb*| = 4.22), and in the HVGIC–antibiotic and HVGIC–multivitamin groups, b* decreased markedly (mean Δb* = −11.66 and −10.50); i.e., the specimens became less yellow rather than more yellow. This is not consistent with simple uptake of the yellow pigment riboflavin present in the multivitamin syrup and may instead reflect surface changes such as opacification or whitening, or changes in the resin coat or the underlying glass ionomer. Dark insoluble precipitates of iron ions have been reported [13,39]; however, the iron preparation produced the lowest mean ΔE_00_ in this study despite having the lowest pH (2.86), indicating that pH alone did not determine the color change. In the antibiotic group, suspending/opacifying particles such as xanthan gum and colloidal silicon dioxide [17] may have contributed to surface opacification, loss of translucency, and a matte appearance, in line with the optical effect of opacifiers reducing light transmission [44]. The high ΔE_00_ value in the antibiotic group appears consistent with the optical and rheological properties of its formulation.

However, the dominant mechanism was not independently verified by surface analysis in our study; therefore, the interpretations above are hypotheses supported by the literature. Although the present study focused on color change, complementary analyses of surface roughness, morphology, and composition may provide a better understanding of whether drug-induced color change is related to pH-induced changes in the restorative surface.

The high viscosity of pediatric medications and the synthetic colorants in their formulations pose a clinical problem in pediatric dentistry, as they increase plaque acidity during prolonged use, thereby creating a cariogenic and erosive environment [20].

In this study, color changes in four restorative materials used in pediatric dentistry were compared after exposure to three pediatric medications frequently prescribed to children, using a twice-daily repeated immersion protocol for one week. The results indicate that the color stability of restorative materials may be related not only to the material’s intrinsic properties but also to the chemical composition of the medication to which it is exposed. In this study, the CIEDE2000 color difference formula (ΔE_00_) was used to evaluate color changes because it is a current method and can detect subtle color differences in dental materials. In our study, these values were evaluated against the perceptibility threshold (PT), updated by Paravina et al. and accepted as the most current reference, of ΔE_00_ = 0.81 (0.34–1.28) and the acceptability threshold (AT) of ΔE_00_ = 1.77 (1.23–2.37) [45]. In our study, the ΔE_00_ value (8.48 ± 3.92) for the HVGIC–antibiotic group was above both thresholds; this indicates a color change that is perceptible and unacceptable at the group-mean level, although statistical significance, perceptibility, and acceptability are distinct concepts.

When evaluated by material type, the highest ΔE_00_ values were recorded in the HVGIC–antibiotic and HVGIC–multivitamin syrup combinations, respectively. This high tendency toward discoloration exhibited by glass ionomer cements may be related to the material’s intrinsic properties and setting mechanism. Indeed, the polyacid matrix content, the microporous structure of the glass particles, and the release of fluoride ions due to fluctuations in the pH of the environment can compromise the material’s stability [14,21]. Similar findings have been described in the literature: in addition to dehydration during the setting and curing stages, the formation of microcracks and pores facilitates chromogen penetration, thereby paving the way for color deterioration [20,46,47]. Because Equia Forte HT was tested together with its resin coat, explanations based on the porosity, microcracks, or matrix degradation of the glass ionomer remain hypotheses and would be relevant only if the coat was damaged or if uncoated surfaces were exposed.

Descriptively, the HVGIC product showed a marked decrease in ΔE_00_ from the antibiotic to the iron preparation, whereas the giomer product changed little across the three medications (Table 4, Figure 3); because the interaction was not significant and the test was underpowered, these cell-level patterns are exploratory. In a study by Çerçi Akçay et al. comparing the Fe^2+^ and Fe^3+^ forms [13], it was reported that the composite showed the lowest ΔE_00_ values, whereas the GIC showed the highest, and that the form of the iron ion may influence the staining dynamics. In the present factorial analysis, the interaction between restorative material and medication type was not statistically significant (p=0.147); however, given the limited power for this term (approximately 68%) and the large observed effect size (partial η^2^ = 0.173), this should not be interpreted as evidence that the effects of material and medication are additive.

In our study, the evaluated giomer product had the lowest mean ΔE_00_ and a comparatively small variation across the three medications, although its difference from the compomer and composite resin products was not statistically significant. This behavior may be related to the S-PRG filler, which has a buffering capacity that may reduce the acidic effect on the material by releasing ions [2,47,48]. This mechanism was not tested in the present study, and the finding applies only to the product studied.

Contrary to our findings, there are studies in the literature reporting that glass ionomer cements exhibit higher color stability and less discoloration than composite resins [23,49,50,51]. In these studies, the authors suggested that, although conventional resin-free glass ionomer cements are hydrophilic, they remain more stable in certain solutions because they do not undergo resin matrix degradation or monomer release. However, the discrepancy between our study and these findings may be explained by variations in the pH values of the pediatric medications tested, differences in immersion protocol durations, brand-specific auxiliary colorants in the medications, and variations in viscosity and acidity levels; these factors may have influenced the magnitude of ΔE_00_ in the specific combinations tested.

In this context, it should be emphasized that HVGIC was evaluated in conjunction with Equia Forte Coat, which is recommended by the manufacturer as an integral component of this restorative system. The literature indicates that resin-based surface coatings, such as Equia Forte Coat, can enhance the surface characteristics of glass ionomer-based materials, reduce pigment penetration and water sorption, and thereby improve color stability [52,53,54]. Indeed, previous studies have demonstrated that coated high-viscosity glass ionomers exhibit delayed discoloration and lower overall color change compared to uncoated specimens, suggesting that the surface coating acts as a protective barrier rather than a stain-absorbing layer [55]. In the present study, the material was tested in strict accordance with the manufacturer’s protocol to accurately replicate routine clinical practice. Nonetheless, the color change observed in the HVGIC group should not be attributed solely to the intrinsic resistance of the underlying glass ionomer substrate, but rather to the overall color stability of the integrated HVGIC system applied according to the manufacturer’s instructions.

Previous studies have shown that iron preparations cause discoloration of teeth and restorations [56]. In this study, despite its low pH, the iron preparation was, contrary to expectations, not the most aggressive staining agent. This finding appears to contradict the results of the study by Kathiria et al. [1], who reported that iron syrups are the strongest staining agents among pediatric medications, and the study by Tüzüner et al. [23]. This discrepancy may be attributed to differences in the forms of iron present in the iron preparations, variations in other components of the formulations, and the composition of the restorative materials used.

In the present study, all restorative materials were evaluated in shade A2 to achieve nominal standardization. Nevertheless, statistically significant differences in baseline L*, a*, and b* coordinates were observed across the material groups prior to immersion. Although ΔE_00_ calculates intra-specimen color shifts relative to each specimen’s own baseline—thereby minimizing the confounding effect of baseline differences on post-exposure comparisons—the CIEDE2000 formula uses the mean lightness, chroma, and hue coordinates within its parametric weighting functions (S_L, S_C, S_H) and rotation term (R_T). Consequently, intrinsic baseline optical disparities may subtly influence the non-linear weighting of chromatic shifts, and this characteristic should be considered when interpreting comparative color stability among distinct restorative material classes.

Notably, the mean ΔE_00_ values in all experimental groups were above the acceptability threshold reported in the literature (this refers to group means and does not imply that every specimen exceeded the threshold). This may be attributable to the duration of repeated exposure to pediatric liquid medications, the medications used and their pH levels, their brands, and differences in the surface roughness of the selected materials. From a clinical perspective, although pediatric dentists frequently consider aesthetic longevity when selecting restorative materials for patients requiring frequent liquid medication regimens, in vitro observations should not be translated directly into prescriptive clinical guidelines. While the Beautifil Kids SA product had the numerically lowest mean ΔE_00_ in this laboratory setting (not significantly different from the compomer and composite resin products), definitive clinical recommendations—such as routine re-application of surface coatings or specific material mandates—warrant verification through controlled long-term clinical trials.

Our study has certain limitations. One of these is the in vitro methodology, which cannot fully reflect the complex and dynamic environment encountered by dental materials within the oral cavity. It is extremely difficult to accurately model, under laboratory conditions, interactions with various agents, including salivary flow, pH fluctuations, and food. Although the total sample size (n=60) conferred robust statistical power (>80%) for detecting significant main effects of both restorative materials and medications, the statistical power for evaluating the material × medication interaction term was approximately 68%; therefore, the non-significant interaction observed in this study should be considered with appropriate caution and validated in larger samples. Levene’s test revealed heterogeneity of variances [F(11,48) = 2.587, *p* < 0.05], driven by differing standard deviations across subgroups. Although a sensitivity analysis using HC3 robust standard errors yielded the same conclusions for the main effects and the interaction, the combination of unequal variances and limited cell size (*n* = 5) warrants cautious interpretation of the results and validation in larger samples.

The fact that the analysis included only three medications and four restorative materials makes it difficult to generalize the findings to all equivalent products available on the market. Each material category was represented by a single commercial product, so product identity and material class are confounded; the findings therefore apply only to the products tested and lay the groundwork for future clinical research. In future studies, it would also be beneficial to investigate the effects of alternative formulations of the medications in question, as well as different oral hygiene procedures, on the discoloration of restorative materials. Furthermore, the absence of an unexposed negative control group stored continuously in artificial saliva or distilled water represents a limitation, as it prevents the complete quantitative isolation of intrinsic material maturation and hydration from medication-induced discoloration. All specimens underwent a 24-h pre-immersion hydration protocol in distilled water prior to baseline measurements to standardize the early water uptake phase. Because water sorption and maturation are material-dependent, they may be confounded with the material effect (particularly for the glass ionomer), and the color changes reported here should therefore be regarded as changes observed after exposure to the medications rather than as changes attributable to the medications alone. Future studies should include, for each restorative product, unexposed negative control groups subjected to the same storage and measurement conditions. In addition, the colorimetric properties of the gray background were not measured, individual repeated readings were not recorded (precluding retrospective calculation of measurement repeatability), no positioning jig was used, and the measurements were not validated against a laboratory-grade spectrophotometer; these factors may have introduced measurement variability.

As an indirect point of reference, the color changes reported for the same material classes after storage in distilled water alone vary widely. After 7 days in distilled water, Dogan and Yıldız reported ΔE_00_ values of 1.85 ± 0.15 for a compomer, 3.05 ± 0.76 for a glass ionomer cement, and 3.78 ± 1.95 for a composite resin [4]. Duzyol et al. reported a ΔE_00_ value of 0.26 ± 0.13 for a compomer after 30 days in distilled water [8], and Choi et al. reported ΔE*_ab_ values of 0.87 ± 0.29, 0.52 ± 0.06, and 0.66 ± 0.34 for a resin composite, a compomer, and a giomer, respectively, after 5 days [10]. The mean ΔE_00_ values of the composite resin, compomer, and giomer products in the present study (2.07–4.36) fall within or close to this range; therefore, the contributions of the medications and of water sorption cannot be separated for these materials. By contrast, the values obtained for the HVGIC product after exposure to the antibiotic (8.48 ± 3.92) and the multivitamin syrup (6.79 ± 2.39) were well above those reported for glass ionomer cements stored in water, suggesting that these changes are unlikely to be explained by water sorption and maturation alone. Because the products, color difference formulas, and protocols differ between studies, this comparison is indirect and does not replace a control group.

Finally, a limitation of the present study is that the integrity of Equia Forte Coat before and after exposure to liquid medications was not microscopically evaluated, nor was the independent contribution of the coating to color change separately quantified, and an uncoated control subgroup was not included in the experimental design. Consequently, it is not possible to definitively separate the relative contributions of the surface resin coat and the underlying restorative bulk substrate to the overall discoloration observed in the HVGIC group. Future studies evaluating HVGIC in both coated and uncoated subgroups would be valuable to clarify the independent and isolated effect of the surface coating on color stability.

Further limitations are that the colors were measured with an intraoral spectrophotometer without a mechanical positioning jig and without verification against a laboratory-grade spectrophotometer; that baseline coordinates differed between materials; that the 7-day exposure was performed without saliva, biofilm, brushing, or thermocycling; and that no surface analysis was performed. Definitive clinical recommendations therefore require controlled clinical studies.

## 5. Conclusions

Considering the limitations of this in vitro study, ΔE_00_ differed significantly among the restorative products and among the three medication exposure conditions, whereas no significant material × medication interaction was detected in a study underpowered for this term. Because no unexposed control group was included, these differences reflect comparisons between exposure conditions rather than the effect of the medications alone. The high-viscosity glass ionomer product (Equia Forte HT with Equia Forte Coat) showed a significantly greater color change than the composite resin, compomer, and giomer products, and exposure to the antibiotic suspension was associated with a significantly greater color change than exposure to the iron preparation. The giomer product (Beautifil Kids SA) had the numerically lowest mean ΔE_00_ but did not differ significantly from the compomer and composite resin products. These findings apply only to the products tested and must be confirmed in controlled, longer-term, and clinical studies.

## Figures and Tables

**Table 1:** Pediatric medications used in the study.

Brand Name	Active Ingredients	Manufacturer	pH	Batch/Lot No.
Augmentin	Amoxicillin trihydrate (equivalent to 400 mg amoxicillin per 5 mL; 8.0% *w*/*v*) and potassium clavulanate (equivalent to 57 mg clavulanic acid per 5 mL; 1.14% *w*/*v*). Xanthan gum (thickener), colloidal silicon dioxide (suspending agent), succinic acid, artificial raspberry/dry flavorings, silicon dioxide (opacifier)	GlaxoSmithKline Pharmaceuticals Industry and Trade Inc., Istanbul, Türkiye	5.70 [27]	25658067A
Polivit	1500 IU Vitamin A, 1 mg Vitamin B, 1.2 mg Vitamin B2, 2 mg Vitamin B6, 7 mg Nicotinamide (PP), 3 mg D-Panthenol, 25 mg Vitamin C, 400 IU Vitamin D3, 5 mg Vitamin E, 3.75 mg Sodium saccharin, 5.00 mg Sodium benzoate Riboflavin (intrinsic yellow pigment), sucrose, liquid glucose, polysorbate 80, sodium saccharin, sodium benzoate, orange essence	Abdi İbrahim Pharmaceutical Industry and Trade Joint Stock Company, Istanbul, Türkiye	4.05 [28]	25D277
Ferro Sanol B	112.50 mg iron (II)-glycine-sulfate complex (equivalent to 20 mg Fe^2+^), 0.43 mg riboflavin-5-sodium phosphate, 0.32 mg vitamin B1 (thiamine hydrochloride), 0.63 mg vitamin B6 (pyridoxine hydrochloride), ascorbic acid, refined sugar, glucose monohydrate, sorbitol, sulfuric acid 95–98%, orange essence, pear essence, deionized water. Riboflavin-5-phosphate (yellow pigment), dark ionic iron matrix, glucose monohydrate, sorbitol, orange/pear essence, sulfuric acid	ADEKA Pharmaceutical Industry and Trade Joint Stock Company, Samsun, Türkiye	2.86 [27]	I059

**Table 2:** Dental materials used in the study.

Product	Material Type	Mixing	Curing	Manufacturer
Charisma Smart	Composite resin	N/A	Light-cure for 20 s	Kulzer GmbH, Hanau, Germany
Nova Compomer	Polyacid-modified composite resin (compomer)	N/A	Light-cure for 20 s	Imicryl, Konya, Türkiye
Beautifil Kids SA	Giomer	N/A	Light-cure for 20 s	Shofu Dental Corporation, Kyoto, Japan
Equia Forte HT	High-viscosity glass ionomer cement	Mix for 10 s	Acid–base setting reaction	GC Corporation, Tokyo, Japan
Equia Forte Coat	Light-curing protective resin varnish	N/A	Light-cure for 20 s	GC Corporation, Tokyo, Japan

**Table 3:** Results of the two-way analysis of variance (two-way ANOVA) for the dependent variable ΔE_00_.

Source of Variation	SS	df	MS	F	*p*	ηp^2^
Corrected model	226.159	11	20.560	5.236	<0.001	0.545
Intercept	845.450	1	845.450	215.311	<0.001	0.818
Material	132.187	3	44.062	11.221	<0.001	0.412
Medication	54.480	2	27.240	6.937	0.002	0.224
Material × medication	39.492	6	6.582	1.676	0.147	0.173
Error	188.479	48	3.927	—	—	—
Total	1260.087	60	—	—	—	—
Corrected total	414.638	59	—	—	—	—

Note: Dependent Variable: ΔE_00_. SS = Sum of Squares (Type III); df = Degrees of Freedom; MS = Mean Square; F = F-statistic; *p* = *p*-value; ηp^2^ = Partial Eta-squared (Effect Size). R^2^ = 0.545 (Adjusted R^2^ = 0.441).

**Table 4:** Comparison of color change (ΔE_00_) values by group.

Material	Antibiotic (*n* = 5)	Multivitamin Syrup (*n* = 5)	Iron Preparation (*n* = 5)	Total (*n* = 15)
Composite resin	4.36 ± 2.28	4.35 ± 2.36	2.07 ± 1.00	3.59 ± 2.15
Compomer	3.78 ± 1.95	2.07 ± 1.30	2.13 ± 0.53	2.66 ± 1.52
Giomer	2.51 ± 2.29	2.76 ± 0.95	2.34 ± 1.00	2.54 ± 1.44
HVGIC	8.48 ± 3.92	6.79 ± 2.39	3.41 ± 1.16	6.23 ± 3.34
Total (*n* = 20)	4.78 ± 3.39	3.99 ± 2.52	2.49 ± 1.04	3.75 ± 2.65 (*n* = 60)

Note: Data are presented as mean ± standard deviation (SD).

**Table 5:** Bonferroni-adjusted pairwise comparisons of ΔE_00_ between materials.

Comparison (I–J)	Mean Difference	SE	Adjusted *p*	95% CI
Composite resin–Compomer	0.927	0.724	1.000	−1.064 to 2.918
Composite resin–Giomer	1.053	0.724	0.914	−0.939 to 3.044
Composite resin–HVGIC	−2.637 *	0.724	0.004	−4.628 to −0.646
Compomer–Giomer	0.125	0.724	1.000	−1.866 to 2.117
Compomer–HVGIC	−3.564 *	0.724	<0.001	−5.555 to −1.573
Giomer–HVGIC	−3.689 *	0.724	<0.001	−5.681 to −1.698

**Note:** Dependent variable: ΔE_00_. * The mean difference is statistically significant at the *p* < 0.05 level (Bonferroni-adjusted). SE, standard error; CI, confidence interval.

**Table 6:** Bonferroni-adjusted pairwise comparisons of ΔE_00_ between medications.

Comparison (I–J)	Mean Difference	SE	Adjusted *p*	95% CI
Antibiotic–Multivitamin syrup	0.790	0.627	0.641	−0.765 to 2.345
Antibiotic–Iron preparation	2.297 *	0.627	0.002	0.743 to 3.852
Multivitamin syrup–Iron preparation	1.507	0.627	0.060	−0.047 to 3.062

**Note:** Dependent variable: ΔE_00_. * The mean difference is statistically significant at the *p* < 0.05 level (Bonferroni-adjusted). SE, standard error; CI, confidence interval.

## Data Availability

The original contributions presented in the study are included in the article, further inquiries can be directed to the corresponding author.

## Abbreviations

The following abbreviations are used in this manuscript:

ANOVA	Analysis of variance
AT	Acceptability threshold
Bis-GMA	Bisphenol A-glycidyl methacrylate
CIE	Commission Internationale de l’Éclairage
CIELAB	International Commission on Illumination $L^{*}a^{*}b^{*}$ color space
CIEDE2000	International Commission on Illumination Color Difference formula 2000
GIC	Glass ionomer cement
HVGIC	High-viscosity glass ionomer cement
LED	Light-emitting diode
PRG	Pre-reacted glass
PT	Perceptibility threshold
PTFE	Polytetrafluoroethylene
SD	Standard deviation
SPSS	Statistical Package for the Social Sciences
S-PRG	Surface pre-reacted glass
TEGDMA	Triethylene glycol dimethacrylate
